# ASD and Genetic Associations with Receptors for Oxytocin and Vasopressin—*AVPR1A, AVPR1B*, and *OXTR*

**DOI:** 10.3389/fnins.2016.00516

**Published:** 2016-11-22

**Authors:** Sunday M. Francis, Soo-Jeong Kim, Emily Kistner-Griffin, Stephen Guter, Edwin H. Cook, Suma Jacob

**Affiliations:** ^1^Department of Psychiatry, University of MinnesotaMinneapolis, MN, USA; ^2^Department of Psychiatry and Behavioral Sciences, University of WashingtonWA, USA; ^3^Biostatistics Shared Resource, Hollings Cancer Center, Medical University of South CarolinaCharleston, SC, USA; ^4^Department of Psychiatry, Institute of Juvenile Research, University of Illinois at ChicagoChicago, IL, USA

**Keywords:** neuropeptides, oxytocin, vasopressin, receptors, social behaviors, repetitive behaviors

## Abstract

**Background:** There are limited treatments available for autism spectrum disorder (ASD). Studies have reported significant associations between the receptor genes of oxytocin (OT) and vasopressin (AVP) and ASD diagnosis, as well as ASD-related phenotypes. Researchers have also found the manipulation of these systems affects social and repetitive behaviors, core characteristics of ASD. Consequently, research involving the oxytocin/vasopressin pathways as intervention targets has increased. Therefore, further examination into the relationship between these neuropeptides and ASD was undertaken. In this study, we examined associations between variants in the receptor genes of vasopressin (*AVPR1A, AVPR1B*), oxytocin (*OXTR*), and ASD diagnosis along with related subphenotypes.

**Methods:** Probands were assessed using Autism Diagnostic Interview-Revised, Autism Diagnostic Observation Schedule, and clinical DSM-IV-TR criteria. Single nucleotide polymorphisms (SNPs) in *AVPR1B* and *OXTR*, and microsatellites in *AVPR1A* were genotyped in ~200 families with a proband with ASD. Family-based association testing (FBAT) was utilized to determine associations between variants and ASD. Haplotypes composed of *OXTR* SNPs (i.e., rs53576-rs2254298-rs2268493) were also analyzed due to previously published associations.

**Results:** Using the additive inheritance model in FBAT we found associations between *AVPR1B* SNPs (rs28632197, *p* = 0.005, rs35369693, *p* = 0.025) and diagnosis. As in other studies, *OXTR* rs2268493 (*p* = 0.050) was associated with diagnosis. rs2268493 was also associated with ASD subphenotypes of social withdrawal (*p* = 0.013) and Insistence on Sameness (*p* = 0.039). Further analyses demonstrated that the haplotype, rs2254298–rs2268493 was found to be significantly associated with diagnosis (A-T; *p* = 0.026). FBAT was also used to analyze *AVPR1A* microsatellites (RS1 and RS3). Both length variants were found to be associated with restrictive, repetitive behaviors, but not overall diagnosis. Correction for multiple comparisons was performed for SNPs tested in each gene region, only *AVPR1B* SNPs remained significantly associated with ASD diagnosis.

**Conclusions:** Autism is a heterogeneous disorder with many genes and pathways that contribute to its development. SNPs and microsatellites in the receptor genes of OT and AVP are associated with ASD diagnosis and measures of social behavior as well as restricted repetitive behaviors. We reported a novel association with ASD and *AVPR1B* SNPs. Understanding of genotype-phenotype relationships may be helpful in the development of pharmacological interventions for the OT/AVP system.

## Introduction

Autism spectrum disorder (ASD) is a heterogeneous disorder that is characterized by impaired social communications and interactions, and restricted, repetitive behaviors (American Psychiatric Association, 2013). The range of clinical presentations suggest multiple etiologies which makes searching for mechanisms and genetic associations challenging. While many genes and pathways have been associated with ASD, the close relationship established between the oxytocin (peptide: OT, gene: *OXT*) and vasopressin (peptide: AVP, gene: *AVP*) systems to social and restricted repetitive behaviors (RRBs) have made these systems a focal point as investigators work to elucidate potential pathophysiological mechanisms and treatments pathways for ASD.

OT and AVP, two closely related neuropeptides, are conserved in both structure and function across mammalian species. While the relationship between these neuropeptide systems and behaviors are complex (Appenrodt et al., 1998; Chang et al., 2012), in general, OT facilitates prosocial and “approaching” behaviors, social memory and recognition, and reduction in anxiety and reaction to stressors (Carter, 1998; Ferguson et al., 2000; Kosfeld et al., 2005; Seltzer et al., 2010; Neumann and Landgraf, 2012). AVP has been associated with animal territoriality and “defensive” behaviors, including sexual cues in human males, social hierarchy, and anxiogenic effects (Landgraf and Wigger, 2003; Guastella et al., 2011; Van der Kooij and Sandi, 2015). It has been noted while closely related, these systems can have opposing physiological effects. OT has primarily parasympathetic actions on the autonomic nervous system, and AVP induces reactions in the sympathetic nervous system and hypothalamic-pituitary-adrenal axis (Sawchenko and Swanson, 1985; Kenkel et al., 2012).

The systems also interact with each other: at high levels the two peptides can act as partial agonists for their homologous receptors (Chini et al., 1996). In a 2014 study of threat response in cats, AVP increased anxiety via the vasopressin receptor 1B and OT increased social affiliation during threat via the vasopressin receptor 1A (Bowen and McGregor, 2014). The receptors of OT and AVP have also been implicated in these behaviors and disorders characterized by impairments in social behaviors including ASD and related subphenotypes (Kim et al., 2002; Wassink et al., 2004; Wu et al., 2005; Lerer et al., 2008; Yang et al., 2010a,b; LoParo and Waldman, 2015). Animal studies which established the involvement of OT and AVP systems in social and repetitive behaviors as well as genetic associations between the neuropeptide systems and disorders with social impairment (i.e., ASD), strengthen the theories to target the OT and AVP systems in the treatment of disorders with social impairments.

Investigators have attempted to influence the oxytocinergic system by directly administering OT. Presently there are over a hundred clinical trials, including 11 in ASD, utilizing intranasal OT (INOT) according to clinicaltrials.gov (as of April 25, 2016). In individuals with ASD, INOT has been shown to increase social interaction (in a simulated ball game task) and improve the identification of emotions (Andari et al., 2010; Guastella et al., 2010). The other part of this equation is the target of OT, the oxytocin receptor (receptor: OTR; gene: *OXTR*). Similar to OT, protein expression of OTR has also been associated with social behaviors including maternal nurturing (Takayanagi et al., 2005). In animal studies, Takayanagi et al. (2005) found *Oxtr*^−/−^ dams had deficits in pup retrieval, a maternal behavior. In addition, research in the last 20 years has found links between *OXTR* and ASD. In a 2005 study, evidence of transmission disequilibrium of two *OXTR* single nucleotide polymorphisms (SNPs; rs2254298 and rs53576) was observed in a Han Chinese ASD sample (Wu et al., 2005). These results were followed up by research in other ethnic populations. Jacob et al. (2007) found an association between rs2254298, but not rs53576, and ASD in a Caucasian sample. In a Japanese sample, Liu et al. (2010) observed significant differences in allelic frequency in rs2254298 between controls and individuals with ASD. In more recent studies, Skuse et al. (2014) investigated associations between 60 tagged *OXTR* SNPs and social recognition skills in ASD families from the UK and Finland. They found rs237887 associated with the Face Recognition Memory Task not only in ASD individuals, but their mothers, fathers and non-affected siblings as well. They also noted an association between rs237865 and diagnosis, but it did not remain after Bonferroni correction. Two recent meta-analyses (LoParo and Waldman, 2015; Kranz et al., 2016) reported associations between *OXTR* and ASD. LoParo and Waldman (2015) reported associations between ASD and the *OXTR* SNPs, rs7632287, rs2268491, and rs2254298 in eight studies and 11 independent samples; analyzing two independent samples and 10 additional studies, Kranz et al. (2016) found rs237889 to be associated with ASD.

Studies exploring the use of intranasally administered AVP (IN-AVP; or similar compound desmopressin, DDAVP) are fewer than INOT, yet the involvement of the AVP system in mammalian social behavior is also important. As mentioned before OT can interact with AVP receptors, making AVP receptors not only a target for AVP and pharmacology that mimic AVP, but for OT agonists as well. Often researchers have examined three nucleotide repeats: two located in the 5′ promoter region (RS1, RS3) and one in the intron (AVR) of *AVPR1A*. In both patient and non-patient populations the lengths of these repeats have been shown to modulate social behaviors including altruistic behavior in healthy adults, prepulse inhibition, and the processing of facial expressions (Knafo et al., 2008; Levin et al., 2009; Zink and Meyer-Lindenberg, 2012; Wang et al., 2016). In an ASD sample, evidence of disequilibrium of the *AVPR1A* microsatellite RS3 was observed in 115 trios (Kim et al., 2002). A few years later, Wassink et al. (2004) found evidence of linkage disequilibrium in an ASD sample as well. In 2010, Yang and colleagues noted an association between ASD and RS1 and RS3 in a Korean sample (Yang et al., 2010b). Tansey et al. (2011) found a significant association between ASD and *AVPR1A* SNPs, but only a weak association with the microsatellites in an Irish sample.

The role of *AVPR1B* has been investigated in some disorders, but not in ASD. Studies have shown that *Avpr1b* knockout mice display decreased ultrasonic vocalization in social situations throughout their lifespans, decreased aggression, and altered social behavior affiliated with dominance (Scattoni et al., 2008; Caldwell et al., 2010; Pagani et al., 2015). The relationship between *Avpr1b* and aggression was strengthened by the Pagani et al. (2015) study, which showed the rescue of aggression by targeted expression of *Avpr1b* in the hippocampus. Additionally, Caldwell et al. (2010) observed increased aggression in *Avpr1b*^−/−^ mice under specific social conditions including competition, food deprivation, and social experience. In humans, SNPs in *AVPR1B* have been linked to child aggression, childhood-onset mood disorders (COMD), prosociality, and autistic traits (Dempster et al., 2007, 2009; Chakrabarti et al., 2009; Zai et al., 2012; Wu et al., 2015). In 2015, Wu et al. observed a significant association between prosociality (mediated through emotional empathy) and *AVPR1B* in a non-clinical Han Chinese male sample. Chakrabarti et al. (2009) noted a nominally significant association between *AVPR1B* and the empathy quotient (EQ) also in a non-clinical sample.

*OXTR, AVPR1A*, and *AVPR1B* are genes that emerged evolutionarily through duplication events and have both paralogous and orthologous relationships in placental mammals (Paré et al., 2016). Given their evolutionary connections, roles in social behavior, and potential as treatment target pathways in ASD, we investigated their genetic variation within an ASD population. *OXTR, AVPR1A*, and *AVPR1B* SNPs or microsatellites were genotyped in ~200 families and analyzed using family based association testing (FBAT). *OXTR* and *AVPR1A* genetic associations have been investigated in ASD previously, but to our knowledge *AVPR1B* variants have not been studied.

## Materials and methods

Subjects were recruited through the Developmental Disorders Clinic of the University of Illinois at Chicago (UIC) Institute for Juvenile Research, referral from providers of autism services, a website providing information about the study, and parent advocacy organizations with the approval of the UIC Institutional Review Board [IRB#: 2007-0239; Title: Interdisciplinary Studies of Insistence on Sameness (IS) in Autism Spectrum Disorders (ASD)]. All participants were provided with a description of the study to obtain informed written consent from adults able to consent for themselves, and parents or guardians of minors and individuals unable to consent prior to their first session.

### Participants

For each participant, a medical history and physical examination were performed by a pediatric neurologist or child psychiatrist and psychiatric evaluation by a child psychiatrist experienced in ASD. Participants were diagnosed using the Autism Diagnostic Observation Schedule (ADOS; Lord et al., 1999; Gotham et al., 2007), Autism Diagnostic Interview-Revised (ADI-R; Rutter et al., 2003; Risi et al., 2006), and confirmed by a physician according to the DSM-IV-TR (American Psychiatric Association, 2000) ASD classification (including autism, Asperger disorder, or pervasive developmental disorder-not otherwise specified (PDD-NOS)). While subjects were not diagnosed with comorbidities, individuals were selected to enrich the IS traits of the sample, which may have increased the probability of comorbidity with certain traits and disorders (i.e., OCD, high anxiety). Additionally, individuals on psychotropic medications were excluded. We performed SNP and microsatellite analysis on two nearly identical samples (Table 1). Two hundred and seven probands including 156 trios and 51 single parent-child duo families were genotyped for SNP analysis. The microsatellite sample consisted of 210 probands of which 206 probands overlapped with the SNP sample (Table 1).

**Table 1:** **Demographic description of the probands analyzed**.

	**SNP analysis**	**MS Analysis**
Number of participants	207	210
Average age (years)	9.89 (±5.48 *SD*)	10.23 (±6.25 *SD*)
Gender	M: 170 (82.1%)	M: 173 (82.4%)
	F: 37 (17.9%)	F: 37 (17.6%)
Race	*N* = 207	*N* = 208
Caucasian/Western European	154 (74.4%)	155 (73.8%)
African American/African	35 (16.9%)	35 (16.7%)
Asian	8 (3.9%)	8 (3.8%)
More than one race	10 (4.8%)	10 (4.8%)
**IQ**		
FSIQ	79.93 (±24.03 *SD*; *N* = 193)	80.70 (±24.49 *SD*; *N* = 194)
VIQ	77.50 (±26.01 *SD*; *N* = 196)	78.41 (±26.64 *SD*; *N* = 197)
NVIQ	82.12 (±23.86 *SD*; *N* = 204)	82.63 (±24.12 *SD*; *N* = 205)

*Demographic data describing the probands utilized for SNP and microsatellite (MS) analyses. The two samples overlap, 206 probands are in both the SNP and microsatellite analyses*.

### Instruments and assessments

The instruments utilized to assess clinical presentation, including social abilities and RRBs, were appropriate for the subjects' ages and abilities.

***Autism diagnostic observation schedule (ADOS)***: The ADOS is a standardized, interactive, semi-structured assessment administered by a trained professional resulting in a standardized score that encompasses social interaction, communication, and restricted interests and repetitive behaviors. The Social Affect domain evaluates communication and reciprocal social interaction including behaviors such as: unusual eye contact, quality of social overtures, initiation of joint attention, amount of facial expression directed at others, and frequency of spontaneous vocalization directed to others gestures (Hus et al., 2014; Hus Bal and Lord, 2015).

***Autism diagnostic interview-revised (ADI-R)***: The ADI-R is a structured interview between a trained professional and the parent or guardian of the individual. The assessment measures behaviors that include reciprocal social interaction, communication and language, and patterns of behaviors. The Insistence upon Sameness domain is comprised of three ADI-R items: (1) compulsions and rituals, (2) resistance to changes in personal routine, and (3) resistance to change in environment (Hus et al., 2007).

***Repetitive behavior scale-revised (RBSR)***: The RBSR assesses the presence, characterization, and severity of RRBs in individuals with developmental disorders (Bodfish et al., 2000). It is a 43-item form that categorizes RRBs into five factors—compulsive behaviors, ritualistic and sameness behaviors, restricted interests, stereotyped behaviors, and self-injurious behaviors (Lam and Aman, 2007).

***Aberrant behavior checklist—community version (ABC-CV)***: The ABC-CV (Aman et al., 1985) is a five-factor, 53-item questionnaire completed by the parent or guardian. The ABC assesses symptoms of irritability and agitation, social withdrawal (lethargy), stereotypic behavior, hyperactivity and non-compliance, and inappropriate speech in individuals aged 6–54 years.

### Genotyping

#### SNPs

We selected two *AVPR1B* and 13 *OXTR* SNPs to genotype based on the literature. DNA was extracted from 10 mL of blood using PureGene® DNA Purification Kit. Next, DNA was quantified with Quant-iT™ PicoGreen® dsDNA Assay (Invitrogen, Carlbad, CA) and the samples normalized to 10 ng/mL. TaqMan® SNP genotyping assays (Applied Biosystems™, Foster City, CA) were then utilized to perform blinded genotyping of the samples. Standard TaqMan® SNP genotyping protocols were used. TaqMan® PCR reactions were performed in 5–2.50 μL Universal Master Mix Amperase® UNG, 0.125 μL TaqMan® probe mix, and 2.375 μL water. PCR conditions (Applied Biosystems™ GeneAmp® PCR System 9700; Foster City, CA) were: OneAmpErase® step at 50.0°C for 2 min, one enzyme activation step at 95.0°C for 10 min, 40–99 alternating cycles of denaturation at 92.0°C for 15 s, and annealing and extension at 58.0°C for 1 min. We used a Roche Light Cycler Model 480-II and Roche Light Cycler 480 Gene Scanning Software v.1.5 (Hoffmann–La Roche AG, Basel, Switzerland) to measure fluorescence intensity of each allele of the final PCR product.

#### Microsatellites

Utilizing primers and protocols from Kim et al. (2002), we genotyped probands, mothers, and fathers blinded to the affect status, family relationship, and demographic data. To summarize, the protocol consisted of multiplex PCR reactions containing 50 ng of DNA, 200 _M dNTPs, 2.5 mM MgCl2, and 0.3 units of AmpliTaq Gold DNA Polymerase (Applied Biosystems™, Foster City, CA) in a 10 μl volume. Microsatellite peaks were measured on the Applied Biosystems 3730xl and sized with Genemapper 3.7 (Applied Biosystems™, Foster City, CA, USA).

### Analysis

Prior to analyzing the SNP data, we checked for genetic Mendelian errors and Hardy-Weinberg equilibrium. Utilizing PLINK v1.07 (Purcell et al., 2007), all but two SNPs were in Hardy-Weinberg equilibrium (*p*-values > 0.05) in the overall sample (note rs237851 and rs2268493: *p* < 0.05 in all parents). Mendelian errors were rare and excluded from analysis on a per SNP basis (the most Mendelian errors were observed in *OXTR* rs11720238 with a total of three). We tested for associations between *OXTR* and *AVPR1B* SNPs, ASD diagnosis, and scores from assessments of social behavior and RRBs. Tests were carried out using *FBAT v2.0.3* (Laird et al., 2000; Rabinowitz and Laird, 2000). FBAT is a non-parametic test of linkage or association between the genotype and the phenotype in which the covariance between the phenotype and offspring genotype is estimated. Tests were conducted assuming an additive inheritance model. Three *OXTR* SNPs (rs53576, rs2254298, and rs2268493) were selected based upon their previous associations with ASD in the literature for haplotype analysis (bi-allelic mode; additive inheritance model). We tested the three marker haplotype and the two marker permutations. The nucleotide repeats of *AVPR1A* were analyzed using a similar methodology. Using FBAT, microsatellite data were checked for Mendelian errors. Mendelian errors were excluded from analysis on a per marker (RS1 and RS3) basis. Next using an additive inheritance model, we used FBAT to examine associations between the genetic variants of *AVPR1A* and ASD diagnosis and other related phenotypes.

## Results

The relationships between genetic variations in the receptor genes of OT and AVP, and ASD diagnosis and ASD-related phenotypes were examined in our analysis. *AVPR1B* SNPs, rs35369693 (*p* = 0.025) and rs28632197 (*p* = 0.006), were associated with ASD diagnosis and remained significant after correction for multiple comparisons for SNPs tested in *AVPR1B* (Table 2A). Next, we explored possible relationships with ASD-related phenotypes. No further associations were noted with *AVPR1B* SNPs (*p*-values were *p* > 0.100 for social communication and restrictive/repetitive subphenotype scores).

Table 2**Significant SNP associations**.

**Table:** 

**A**			
**SNP**	***p*-value**	**Z**	**Risk Allele**
AVPR1B rs35369693^*^	0.025	2.24	G
AVPR1B rs28632197^*^	0.006	2.77	A
OXTR rs2268493	0.050	1.96	T

**Table:** 

**B**						
**Phenotype**		**SNP**	***p*****-value**	**Z**	**Allele**	**Risk Allele**
Social communications	ABC—Social withdrawal	OXTR rs4686302	0.036	–2.09	T	C
		OXTR rs2268493	0.013	–2.49	C	T
	ADOS—Social affect	OXTR rs2254298	0.023	2.27	A	A
Restricted/Repetitive behaviors	ABC—Stereotypy	OXTR rs4686302	0.018	2.37	C	C
	ADOS—RRB	OXTR rs1042778	0.011	–2.54	T	G
	ADI—IS	OXTR rs2268493	0.039	–2.07	C	T

*(A) list the SNPs in both OXTR and AVPR1B that were found to be significantly associated with ASD diagnosis. (B) notes the SNP significantly associated with ASD-related phenotypes, mainly social communications and RRBs, in our sample using the additive inheritance model. None of the AVPR1B SNPs were significantly associated with the different subphenotypes (p > 0.100). The remaining OXTR SNPs were also not significantly associated with the subphenotypes (p > 0.054)*.

* *denotes significant after correcting for multiple comparisons*.

*OXTR* rs2268493 (*p* = 0.050) was associated with ASD diagnosis, but did not remain significant after multiple comparison correction for the number of *OXTR* SNPs that were tested. We also examined ASD-related subphenotypes and found associations with Several *OXTR* SNPs (Table 2B). rs2268493 was not only associated with ASD diagnosis, but with measures of social behaviors as well as RRBs (ABC-Social Withdrawal: *p* = 0.013; ADI-R IS as a restricted behavior: *p* = 0.039). Similarly, rs4686302 was another SNP found to be significantly associated with both subphenotype categories within ASD (ABC-Social Withdrawal: *p* = 0.036; ABC-Stereotypy: *p* = 0.018). We also found rs2254298 to be significantly associated with ADOS-Social Affect (*p* = 0.023). No further associations were noted for the remaining *OXTR* SNPs and the characterized subphenotypes (*p*-values were *p* > 0.054). None of the subphenotype associations were significant after correcting for multiple comparisons. Additionally, we performed a haplotype analysis utilizing FBAT. Both rs53576 and rs2254298 have been actively studied variants in the ASD literature (Wu et al., 2005; Jacob et al., 2007; Lerer et al., 2008; Liu et al., 2010; Wermter et al., 2010; Campbell et al., 2011; Di Napoli et al., 2014), therefore, they were used along with rs2268493 to create our haplotype. Haplotype analysis yielded significant associations with ASD diagnosis and both ASD-related phenotypes (Table 3). The two marker haplotype rs2254298–rs2268493 (A-T: *p* = 0.026) was significantly associated with ASD diagnosis. This same haplotype was significantly associated with social behaviors as measured by ADOS (Social Affect: *p* = 0.016) and ABC (Social Withdrawal: *p* = 0.036). Overall, haplotypes were associated with ASD diagnosis, RRBs as assessed by ADOS, and social behaviors measured by ADOS and ABC.

**Table 3:** **Haplotype analysis using three markers from ***OXTR*****.

**Phenotype**	**Markers**			**Haplotype**			***p*-value**	**Z**
ASD		rs2254298	rs2268493		A	T	0.026	2.23
ADOS−-Social affect	rs53576	rs2254298	rs2268493	G	A	T	0.008	2.63
	rs53576	rs2254298		G	A		0.017	2.40
		rs2254298	rs2268493		A	T	0.016	2.41
ABC−-Social withdrawal	rs53576	rs2254298	rs2268493	G	G	C	0.009	−2.59
	rs53576		rs2268493	G		C	0.009	−2.62
		rs2254298	rs2268493		A	T	0.036	2.10
ADOS−-RRB	rs53576	rs2254298	rs2268493	A	G	C	0.032	−2.15
	rs53576		rs2268493	A		C	0.024	−2.25

*Haplotypes were created from three SNPs (rs53576, rs2254298, and rs2268493) previously associated with ASD. Data were analyzed for associations with ASD diagnosis and subphenotypes*.

FBAT was also utilized to examine relationships between microsatellites in *AVPR1A, ASD*, and subphenotypes. No main effects were noted; however, significant associations were observed with assessments measuring RRBs. Length variants in both RS1 and RS3 were found to be significantly associated with different measures of RRBs (Table 4A). Some studies have categorized the variant lengths as short (S) and long (L) due to the variation of defining lengths across studies (i.e., RS1 306 was analogous to RS1 308 and RS1 312 in different studies—see Table 2 in Kantojärvi et al., 2015). While we analyzed the microsatellite data using individual lengths, afterwards for ease of comparison with other published studies we categorized the length variant values as S or L. The cutoff was determined by creating two approximately equal groups (Knafo et al., 2008). The split occurred between the highest and second highest frequencies reported in our sample (Table 4B). Utilizing S/L categories, we noted long variants of RS1 and RS3 were associated with different measures of RRBs in our sample. We found a significant association with a short length variant (RS1 S) and RRBs as measured by the RBSR.

Table 4**FBAT analysis for ***AVPR1A*** microsatellites and allelic frequencies**.

**Table:** 

**A**					
		**Marker**	**Allele**	***p*****-value**	**Z**
Restricted/Repetitive behaviors	ABC–Stereotypy	RS1	318	0.015	2.44
		RS1	322	0.027	–2.21
	ADOS–RRB	RS3	344	0.003	2.93
	RBSR–Compulsive and Ritualistic/Sameness subscale total	RS1	310	0.020	–2.32
		RS3	336	0.014	–2.45
	RBSR–Total	RS1	310	0.047	–1.98
		RS3	336	0.008	–2.65
		RS3	338	0.042	–2.03

**Table:** 

**B**			
**RS1**		**RS3**	
**Allele**	**Percent**	**Allele**	**Percent**
306	0.005	317	0.012
310	0.100	326	0.003
**314**	**0.387(S)**	328	0.063
318	0.263(L)	330	0.104
322	0.106	**332**	**0.236(S)**
326	0.079	334	0.195(L)
330	0.023	336	0.130
334	0.032	338	0.110
338	0.006	340	0.041
342	0.001	342	0.015
		344	0.046
		346	0.029
		348	0.015
		350	0.002
		353	0.001

*(A) lists the significant associations with ASD-related phenotypes. (B) displays allelic frequencies of AVPR1A in our sample. The bold text denotes the allele with the highest frequency. The underlined text identifies the second highest allelic frequency for the markers. This “border” is often used to delineate short (S) and long (L) allelic categories. The categories are determined by creating approximately equal percentages in each category (Knafo et al., 2008)*.

## Discussion

The core symptoms of ASD include impairments in social communication and the presence of RRBs. Given the well-established links between the OT/AVP system and these behaviors, genetic variants and associations were investigated in this ASD sample. We used FBAT to examine associations between receptor genes of these neuropeptides and ASD. The variants in *AVPR1B* (SNPs—rs35369693 and rs28632197), *AVPR1A* (two microsatellites—RS1 and RS3), and *OXTR* (13 SNPs) were studied in association with ASD diagnosis, as well as scores from assessments measuring deficits in social behaviors and RRBs.

To our knowledge, there are no published studies reporting *AVPR1B* associations with ASD. Our results showed that both *AVPR1B* SNPs, rs35369693, and rs28632197, were significantly associated with ASD diagnosis (Table 2A). Variants within *AVPR1B* have been linked to social behavior in humans and other mammals. As mentioned earlier, *Avpr1b*^−/−^ mice show decreased aggression and altered dominance behavior (Caldwell et al., 2010; Pagani et al., 2015). Human studies including our results have linked *AVPR1B* to disorders with social components including bipolar type I, depression, autistic traits as measured by EQ, childhood aggression, COMD, suicidal attempts, prosociality, and emotional empathy (Dempster et al., 2007; Chakrabarti et al., 2009; Leszczynska-Rodziewicz et al., 2012; Zai et al., 2012; Szczepankiewicz et al., 2013; Luppino et al., 2014; Wu et al., 2015). Specifically, Zai et al. (2012) found rs3536969C to be underrepresented in 177 aggressive child cases as compared to adult matched controls. This finding remained significant when a homogenous European Ancestry subsample was analyzed. In a Hungarian sample (382 nuclear families), Dempster et al. (2007) found rs35369693 to be significantly associated with COMD. They also noted a sex difference: when divided by sex, the association remained significant for affected females. Additionally, associations between anxiety and panic disorders and *AVPR1B* rs28632197 have been observed (Keck et al., 2008). Keck and colleagues compared individuals with panic disorder with matched controls and found nominal associations with panic disorder and *AVPR1B*, including rs28632197 (*p* = 0.046), in their main sample (not significant in their replication sample or the combined sample). They also examined interactions between *AVPR1B* and *CRHR1* in their samples. Two SNP pairs, *AVPR1B* rs28632197—*CRHR1* rs878886 and *AVPR1B* rs28632197—*CRHR1* rs187631, were also found to be significantly associated with panic disorder. While previous findings were not conducted in an ASD sample, anxiety, aggression, and depression are often co-morbid issues along with restrictive behaviors and social challenges in ASD (Giles and Martini, 2016; Russell et al., 2016).

The *OXTR* SNP, rs2268493, was not only related to diagnosis, but also with scales measuring impairments in social behaviors and RRBs (Tables 2A,B). Both subphenotypes, social behaviors and RRBs, are characteristic of ASD. While these findings did not withstand correction for multiple comparisons, they replicated similar findings in the literature. Yrigollen et al. (2008) found rs2268493 to be significantly associated with diagnosis, communication skills, and stereotyped behaviors. Subsequently, Campbell et al. (2011) used a narrow diagnosis of autism in an Autism Genetic Resource Exchange (AGRE) sample and found rs2268493T to be significantly associated with their diagnosis criteria. In schizophrenia, rs2268493T has also been associated with social cognition (Davis et al., 2014). Decreased activity in the mesolimbic reward circuitry during reward anticipation in typically-developed adults was associated with the T homozygotes of rs2268493 (Damiano et al., 2014). Additionally, as part of a haplotype rs2268493 was associated with Asperger Syndrome (Di Napoli et al., 2014).

Other significant associations were noted between variants in *OXTR* and assessments of core characteristics of ASD in the entire sample (Table 2B). Besides rs2268493, which was associated with social behaviors and RRBs, rs4686302 had significant associations across both categories of phenotypes. rs4686302 has been associated with emotional empathy in a non-clinical Chinese sample (Wu et al., 2012). Wu et al. (2012) found the CC homozygotes to have less cognitive and trait empathy than the CT heterozygotes in their sample. We found rs1042778 was significantly associated with RRBs as measured by ADOS. This SNP (G-allele) has also been associated with ASD diagnosis in an AGRE sample (Campbell et al., 2011) and creative cognition in a non-patient Han Chinese sample (De Dreu et al., 2014). In the study conducted by De Dreu et al. (2014), creativity gives the individual the ability to adapt and be flexible to changing circumstances and social situations.

A significant association was also observed between rs2254298 and ADOS-Social Affect. This SNP has been affiliated with social impairment in two recent studies, Parker et al. (2014) found rs2254298 to be associated with social impairment in an ASD and typically developing sample. Also, in a meta-analysis conducted by LoParo and Waldman (2015) they found rs2254298 and two other *OXTR* SNPs to be associated with ASD in analysis of eight studies and 11 independent samples. In 2008, Lerer et al. found rs2254298 to be associated with Vineland Adaptive Behavior Scales-2nd Edition (VABS-II) and an active marker in their haplotype analysis in a male ASD Israeli sample (Lerer et al., 2008). Given these previous ASD findings and the relationship between diagnosis and both categories of subphenotypes in our sample, rs2254298 and rs2268493 in addition to rs53576 were used in our haplotype analysis. rs53576 has had previous associations in ASD samples. rs53576 was found to be associated with ASD, social behavior, and emotional withdrawal in a patient population (Wu et al., 2005; Chang et al., 2014; Haram et al., 2015). However, in this sample we did not find associations with rs53576 as an individual SNP. The two marker haplotype, rs2254298A–rs2268493T was found to be associated with ASD diagnosis (Table 3).

In addition to SNPs, we studied the microsatellites located in the 5′ flanking region of *AVPR1A*, RS1, and RS3, in association with ASD-related phenotypes (Table 4A). Our significant associations were observed with several measures of RRBs. *AVPR1A* RS3 length variants that can be categorized as L were associated with these assessments. While we did not find associations with diagnosis or social behavior in the whole sample, others have found affiliations in ASD samples (Kim et al., 2002; Wassink et al., 2004; Yang et al., 2010b; Kantojärvi et al., 2015). Associations between *AVPR1A* microsatellites and social behaviors were also noted in both patient and non-patient samples in several studies (Knafo et al., 2008; Ebstein et al., 2009; Levin et al., 2009; Meyer-Lindenberg et al., 2009).

Future research should address the limitations of our study along with the inconsistencies in the literature. Firstly, our findings (SNPs, haplotypes, and microsatellites) need to be replicated in a larger sample, particularly given the small effect of specific inherited common variants in ASD based on genome-wide association studies of ASD (Anney et al., 2012; Chaste et al., 2015). A larger sample would also allow additional analysis of other subgroups, such as sex differences. This is of interest, because there are sex differences between the OT and AVP systems and ASD is diagnosed in males more often than females (Chakrabarti and Fombonne, 2005; Carter et al., 2008). Another factor that can contribute to inconsistent results is the use of differing diagnostic tools and criteria, especially because of the heterogeneity of the disorder. Alternative tools in both international and national studies could be measuring slightly different aspects of behaviors. Lastly, varying methodologies can be a contributing factor as well. In the analysis of microsatellites, differing groups define the variant lengths differently (see Table 2 in Kantojärvi et al., 2015).

OT and AVP systems influence mammalian brain pathways related to social and adaptive (vs. rigid, restricted) behaviors. There is a growing literature about OT and AVP in a range of disorders including schizophrenia, COMD, bipolar disorder as well as neurodevelopmental disorders (Dempster et al., 2007; Leszczynska-Rodziewicz et al., 2012; Davis et al., 2014; Francis et al., 2014). There are currently many clinical trials exploring the therapeutic value of OT and AVP (e.g., in ASD, schizophrenia, major depressive disorder, and substance dependence). With shifts from disorder-based to circuit-based therapeutic targets, studies of OT and AVP genetics need to explore associations with social and rigid behavior subphenotypes in larger samples. Overall, our results suggest further research of the OT/AVP system, including *OXTR, AVPR1A*, and especially *AVPR1B* in ASD and other disorders with social impairment and/or RRBs.

## Author contributions

Genotyping, analysis, and manuscript preparation were performed by SF and SJ. EK contributed to genetics data analysis throughout the study. SK assisted with microsatellite methods and analyses. SG assisted with phenotype data collection and data management. EC contributed to sample collection and to manuscript preparation. SJ was the principal investigator for the study and coordinated the project. All authors read and approved of the final manuscript.

### Conflict of interest statement

SJ has been a site-investigator for a Roche multisite ASD clinical trial, a site-investigator for a federally funded oxytocin ASD clinical trial, and a consultant for Genentech. EC has been a consultant for a Seaside Therapeutics multisite clinical trial. SK serves as part of Pfizer, Shire, Roche, and Ironshore Pharmaceuticals clinical trials. For the remaining authors there are no conflicts of interest.

## Abbreviations

RRB(s): Restrictive/Repetitive Behavior(s)
SNP(s): Single Nucleotide Polymorphism(s).
